# Determinants of echolocation call frequency variation in the Formosan lesser horseshoe bat (*Rhinolophus monoceros*)

**DOI:** 10.1098/rspb.2009.1185

**Published:** 2009-08-19

**Authors:** Shiang-Fan Chen, Gareth Jones, Stephen J. Rossiter

**Affiliations:** 1 School of Biological Sciences, University of Bristol, Bristol BS8 1UG, UK; 2 Conservation and Research Center, Taipei Zoo, Taipei 11656, Taiwan, Republic of China; 3 School of Biological and Chemical Sciences, Queen Mary, University of London, London E1 4NS, UK

**Keywords:** Chiroptera, acoustic, microsatellites, cultural drift, maternal transmission

## Abstract

The origin and maintenance of intraspecific variation in vocal signals is important for population divergence and speciation. Where vocalizations are transmitted by vertical cultural inheritance, similarity will reflect co-ancestry, and thus vocal divergence should reflect genetic structure. Horseshoe bats are characterized by echolocation calls dominated by a constant frequency component that is partly determined by maternal imprinting. Although previous studies showed that constant frequency calls are also influenced by some non-genetic factors, it is not known how frequency relates to genetic structure. To test this, we related constant frequency variation to genetic and non-genetic variables in the Formosan lesser horseshoe bat (*Rhinolophus monoceros*). Recordings of bats from across Taiwan revealed that females called at higher frequencies than males; however, we found no effect of environmental or morphological factors on call frequency. By comparison, variation showed clear population structure, with frequencies lower in the centre and east, and higher in the north and south. Within these regions, frequency divergence was directional and correlated with geographical distance, suggesting that call frequencies are subject to cultural drift. However, microsatellite clustering analysis showed that broad differences in constant frequency among populations corresponded to discontinuities in allele frequencies resulting from vicariant events. Our results provide evidence that the processes shaping genetic subdivision have concomitant consequences for divergence in echolocation call frequency.

## 1. Introduction

Intraspecific geographical variation in vocalizations has been documented in a range of animal groups, including birds (Soha *et al.* 2004; Wright *et al.* 2005), mammals (Mitani *et al.* 1992) and invertebrates (Eiriksson 1992). However, the origin and maintenance of such variation is not clear, with most hypotheses emphasizing the roles of population history (vicariance) or reduced dispersal because of local adaptation. Vicariance-based explanations reason that populations undergo cultural divergence in isolation due to drift and/or selection, and show incomplete homogenization on secondary contact (Grant *et al.* 2000). Adaptation-based models postulate that variation arises via adaptation to different environments and thus exchange between populations exhibiting different vocal characteristics will be hampered because immigrants will be ill-suited to vocal communication in their new habitat (Slabbekoorn & Smith 2002*a*). Extending this scenario, it has been suggested that variation in vocal signals can promote parapatric population divergence, reproductive isolation and, ultimately, ecological speciation (see discussion in Slabbekoorn & Smith 2002*b*).

Both explanations are expected to lead to concordance between variation in call and neutral genetic variation. Trends between genetic subdivision and variation in vocalizations have to date been studied mostly in birds and humans, both of which exhibit cultural vocal learning. In birds, the expectation that dialect boundaries should correlate with genetic discontinuities has received mixed empirical support (reviewed in Slabbekoorn & Smith 2002*a*). Poor correspondence has been found in parrots, for example, attributed to the continuation of vocal learning after dispersal (Wright & Wilkinson 2001; Wright *et al.* 2005). By comparison, it has been suggested that the vertical transmission of cultural attributes in humans means that similarities in language will reflect common ancestry; however, data are once again equivocal. Cavalli-Sforza (1997) found broad agreement between linguistic and genetic trees, and others have suggested that language affiliation might actually cause and maintain genetic differentiation among populations (Barbujani *et al.* 1996). Others have found little concordance between genetic and linguistic structure, possibly due to language replacement outpacing gene replacement via horizontal learning (Hunley & Long 2005).

Bats use vocalizations to orient in space, and often for the detection, localization and classification of prey (Griffin 1958). Although sonar signals are not functionally equivalent to animal vocalizations such as bird song, which have evolved specifically for communication (Barclay 1999), they are nonetheless subject to some comparable selection pressures. Call frequency is inversely related to wavelength, and short wavelengths are necessary for obtaining strong echoes from small targets (Houston *et al.* 2004). Call frequency might therefore influence a bat's ability to detect targets of a given size. Bats alter their echolocation frequency in relation to habitat (Obrist 1995; Wund 2006), and geographical variation is documented in several species (Barclay *et al.* 1999; Guillén Servent *et al.* 2000; O'Farrell *et al.* 2000; Law *et al.* 2002; Davidson & Wilkinson 2002; Aspetsberger *et al.* 2003; Macias & Mora 2003; Gillam & McCracken 2007). Biosonar signals are also known to vary among individuals (Fenton et al. 2004), are altered in the presence of foraging conspecifics (Obrist 1995; Ratcliffe *et al.* 2004; Hiryu *et al.* 2006) and can influence the behaviour of other bats (Fenton 2003). Furthermore, echolocation calls are also used in communication (Kanwal *et al.* 1994; Ma *et al.* 2006; Melendez *et al.* 2006). Bats can locate foraging conspecifics (Barclay 1982) and roosts (Ruczyński *et al.* 2007) by eavesdropping on echolocation calls, and acoustic character displacement occurs so that horseshoe bat species often maintain ‘private bandwidths’ of call frequencies that minimize overlap with other species (Russo *et al.* 2007).

Bats in the families Rhinolophidae and Hipposideridae produce echolocation pulses dominated by a constant frequency (CF) component (Neuweiler 2000), which are adapted to detect the acoustic glints produced by insect wing beats (Neuweiler 1989; Schnitzler & Kalko 1998). This simple structure belies a sophisticated control system (Pollak & Casseday 1989) in which call frequency can be adjusted within a narrow receiving range in response to echo feedback during flight (Doppler-shift compensation) (Schuller & Pollak 1979; Trappe & Schnitzler 1982). Horseshoe bats (genus *Rhinolophus*) represent an ideal model system for studying the determinants of geographical variation in vocal signals for three main reasons. First, the CF component of the call can be measured accurately to within 1 kHz and so assessing call variation does not rely on qualitative comparisons of spectrograms, as is the case for many animal vocalizations. Second, cultural learning has previously been established in this genus, with the fine-tuning of call frequency determined in part by vertical transmission from mother to offspring (Jones & Ransome 1993). Third, communication calls that incorporate the CF component of the echolocation signal have been described in both captive (Ma *et al.* 2006) and wild colonies of *R. ferrumequinum* (Andrews & Andrews 2003; Andrews *et al.* 2006), and also appear to function in mother–young communication (Matsumura 1981). Kingston & Rossiter (2004) found that in recently diverged sympatric populations of *R. philippinensis*, positive assortative mating correlates with echolocation call frequency. Thus, even where frequency variation is too slight to affect sensory ecology, it might still have an impact on vocal communication if these call types are correlated (Kingston *et al.* 2001; Kingston & Rossiter 2004).

In addition to maternal effects, intraspecific CF call variation has been found to correlate factors such sex, age, body condition and forearm length (Jones *et al.* 1992; Jones 1995; Guillén Servent *et al.* 2000; Siemers *et al.* 2005; Armstrong & Coles 2007) as well as the size of morphological characters that are directly involved in either sound production (Armstrong & Coles 2007) or reception (Francis & Habersetzer 1998). However, such trends are not always supported (see discussion in Armstrong & Coles 2007). Climatic variables such as humidity, precipitation and temperature can also be important; for example, call frequency correlates positively with body temperature (Huffman & Henson 1993), while a negative association with humidity detected in *Hipposideros ruber* might be due to the attenuation of higher frequencies in moist air (Guillén Servent *et al.* 2000). Despite increasing numbers of reports of variation in echolocation calls, little is known about how this relates to genetic structure. A recent study of echolocation call variation in *Rhinolophus cornutus* showed a bimodal distribution of mean call frequency in the face of gene flow, and suggested a ‘maternal transmission’ hypothesis (Yoshino *et al.* 2008). Here we examine the basis of call frequency variation in the Taiwanese endemic *Rhinolophus monoceros* by assessing the relative importance of morphological, environmental, geographical and genetic variables. Following Sokal (1988), we predict that due to vertical learning in this genus, similarities in call frequencies will reflect common ancestry. We therefore hypothesize that CF differences will correlate with genetic differentiation based on neutral markers due to an underlying pattern of genetic isolation-by-distance. Alternatively, if call frequency is locally adapted regardless of gene flow, then vocal divergence will not necessarily correspond to genetic subdivision but might instead correlate with environmental factors. Finally, if variation in call frequency is also a consequence of vicariant events, we predict that any discontinuities in allelic frequencies will also correspond to divisions among the patterns of CF variation.

## 2. Material and methods

### (a) Field sampling

**Figure 1.**
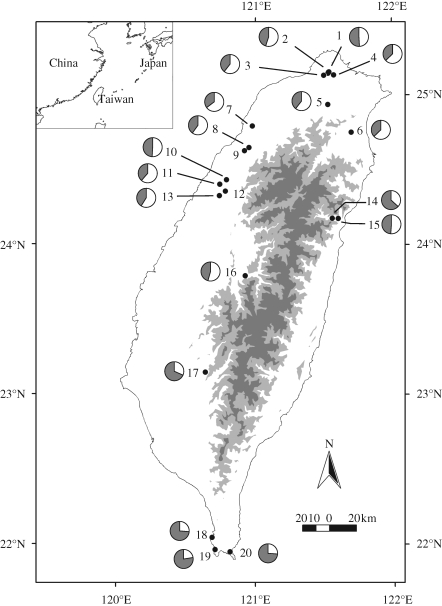
Map of Taiwan showing sampling localities of *Rhinolophus monoceros* populations analysed in this study. Population codes are the same as in the electronic supplementary material. Light grey and dark grey shading indicate zones of over 1000 and 2000 m above sea level, respectively. For each population, the average membership of the two clusters inferred by STRUCTURE is shown as a pie-chart, with cluster 1 as white and cluster 2 as grey.

Bats were captured between June 2002 and September 2003 at 20 roosts (hereafter referred to as populations 1–20) across Taiwan (figure 1). For each individual captured, we recorded its sex, age (juvenile/ adult), reproductive status, forearm length and body mass.

### (b) Echolocation call recording

Bats were recorded in the summer (June–October). Additionally, individuals from seven populations (1–4, 14, 18–19) were recorded in the winter (January–March) to allow seasonal comparisons. Juveniles captured at one population (2) were used to compare call frequencies between adults and juveniles. Pregnant females were excluded from analyses.

For echolocation call recording, bats were held 30 cm from a microphone attached to a D980 Pettersson Elektronik bat detector (Pettersson Elektronik AB, Sweden). Time-expanded (10 × ) calls were recorded onto a Sony WM-D6C cassette recorder. Echolocation calls were analysed using the sound analysis software BatSound Pro (Pettersson Elektronik AB, Sweden). The maximum energy (in kHz) of the dominant (second) harmonic of each CF call was determined from a power spectrum of a call. A 4096-point fast Fourier transform (FFT) and a Hanning window were used within a 5 kHz frequency range to give a frequency resolution of 64 Hz. We checked for call frequency variation within individuals by comparing 10 randomly chosen calls for 20 bats. Inter-individual variation was small, typically around 0.2 kHz (standard deviations ranging from 0.035 to 0.098 kHz). Therefore, single calls per individual were used in subsequent analyses.

### (c) Non-genetic determinants of echolocation call frequency variation

We tested a range of potential determinants of echolocation call frequency. In addition to population location and sex, we studied three morphological variables: forearm length (mm), body mass (g) and a standard index of adult body condition defined as the residual in a linear regression of body mass versus forearm length (e.g. Schulte-Hostedde *et al.* 2001). We also identified three environmental variables: the local annual means for temperature and relative humidity, and elevation. Temperature and humidity values were obtained from the nearest meteorological station (mean distance to roost: 3.88 ± 2.01 (s.d.) km, range 1.05–7.99 km, *n* = 20). A longer-term study of environmental conditions at three sites revealed little seasonal variation (S.-F. Chen, G. Jones & S. J. Rossiter 2002–2003, unpublished data). We predicted high humidity would be associated with lower frequencies, because atmospheric attenuation increases with humidity and frequency. Bats would therefore need to call at lower frequencies at higher humidity levels to get similar echo strengths from a given target (Bazley 1976). Humidity and temperature have been shown to correlate with call frequency in other bat species (Huffman & Henson 1993; Guillén Servent *et al.* 2000). Elevation varies across sampling localities, and is expected to influence humidity. Meteorological data were obtained from the Central Weather Bureau, Taiwan and elevation information from a 1∶25 000 topographic map.

We first tested for an effect of age using a *t*-test to compare adults and offspring from population 2. To assess for seasonal differences across seven populations we used a general linear model (GLM) in Minitab (Minitab Inc.). To test for an effect of geographical location and sex, we tested for differences in call frequency among localities and between sexes using a GLM in which both variables were treated as factors. Call frequency data collected from both sexes were tested for normality using an Anderson–Darling test.

To assess the impact of other non-genetic determinants of call frequency, we constructed a generalized linear mixed model (GLMM) using the software S-PLUS (Insightful Inc.). Morphological (forearm length, body mass and index of body condition) and environmental variables (temperature, relative humidity and elevation) were fitted as fixed effects whereas population identity was coded as a random effect. This approach overcomes the potential problem of non-independence among measurements that are spatially correlated (i.e. from the same population).

### (d) Genetic structure and echolocation call frequency variation

To test for a relationship between neutral genetic structure and call frequency, we analysed multi-locus microsatellite data. Wing-membrane biopsy punches (3 mm diameter) of recorded individuals were taken and stored in ethanol (see Chen *et al.* 2006), and genomic DNA was isolated and genotyped at six microsatellite markers (see Chen *et al.* 2008 for details).

The relationship between neutral genetic structure and call frequency was assessed using two approaches. First we tested for a correlation between call frequency divergence and genetic divergence among populations. We plotted pairwise *F*_ST_/(1 − *F*_ST_) values against corresponding mean pairwise call frequency differences, analogous to an isolation-by-distance model (genetic divergence versus geographic distance). The correlation coefficient was derived using a Mantel test and significance was tested by permutation (10 000 times) in Arlequin (Excoffier *et al.* 2005). Because isolation-by-distance has previously been reported for the Taiwanese population of *R. monoceros* (Chen *et al.* 2008), we also applied a partial Mantel test to examine the correlation between call frequency and genetic divergence after correcting for the effect of geographical distance. Pairwise linear Euclidean distances (km) between localities were computed from geographical coordinates.

To identify potential discontinuities in allele frequencies without reference to sampling locality, which might also correlate with variation in call frequency, we applied the Bayesian clustering method using the program Structure (Pritchard *et al.* 2000). This method infers the most probable number of clusters (*K*) in the data by assigning individuals to population groupings so that linkage-disequilibrium in the dataset is reduced. We used a burn-in length of 20 000 and a run length of 1 million without prior population information, and undertook 20 independent runs for each *K* from 1 to 8. To estimate the number of clusters present in the data we inspected the value of *K* that maximized the posterior probability of the data, given by *p*(*K*|X) and also derived values of Δ*K*, defined as the mean of the absolute value of the second-order rate of change of *L*(*K*) with respect to *K* divided by the standard deviation of *L*(*K*). This value has been shown to be useful in detecting the number of clusters present where *L*(*K*) increases monotonically (Evanno *et al.* 2005).

The outcomes of independent runs for the most likely value of *K* were then sorted based on their pairwise similarity (*G*) following the method outlined by Jakobsson & Rosenberg (2007). This was undertaken in the software Clumpp with the *FullSearch* algorithm. Pairs of runs that yielded similarity scores of less than 0.95 were removed.

## 3. Results

### (a) Non-genetic determinants of echolocation call frequency variation

We recorded 554 individuals of *Rhinolophus monoceros* comprising 240 adult females, 296 adult males and 18 juveniles of both sexes (see appendix 1 in the electronic supplementary material for details). Females were significantly larger than males according to both forearm length (*t*_484_ = 9.70, *p* < 0.01, females: 37.89 ± 1.04 (s.d.) mm; males: 37.02 ± 1.00 mm) and body mass (*t*_484_ = 2.72, *p* < 0.01, females: 4.91 ± 0.51 g; males: 4.80 ± 0.49 g).

An Anderson–Darling test showed that distributions of call frequency did not deviate from normality for all sex and age combinations. Adult males and females showed significantly higher frequencies than juveniles of the same respective sex (two-tailed *t*-test: females: *t*_55_ = 5.40, *p* < 0.001; males: *t*_22_ = 5.13, *p* < 0.001). Call frequency did not fluctuate between summer and winter (GLM, *F*_1,304_ = 0.16, *p* = 0.686) and, therefore, samples from summer and winter were pooled for subsequent analyses.

**Figure 2.**
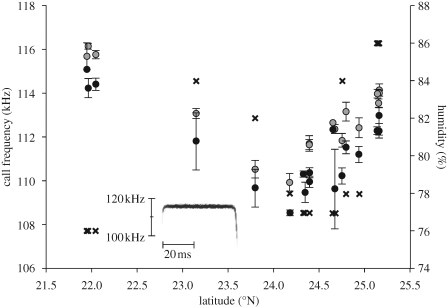
Mean echolocation call frequency with standard errors for female (grey circles) and male (black circles) *Rhinolophus monoceros* sampled from 20 populations across Taiwan. Populations are ordered according to their latitude (see figure 1). Relative humidity data points are presented as crosses. The inset (lower left) shows a spectrogram for a typical *R. monoceros* echolocation call.

Mean population echolocation call frequency varied markedly across Taiwan in both sexes (figure 2). A GLM revealed that significant variation in call frequency was explained by geographical location, as revealed by the effect of population (*F*_19,515_ = 57.76, *p* < 0.001). Central and eastern populations consistently displayed lower call frequency compared to their more northern and southern counterparts. The highest average divergence between populations was 6.22 kHz in females and 6.53 kHz in males, although individuals showed greater differences (10.47 kHz in females and 8.76 kHz in males). The effect of sex itself was also highly significant (*F*_1,515_ = 181.90, *p* < 0.001): females called at higher frequencies than males (females: 113.72 ± 1.84 kHz; males: 111.77 ± 1.85 kHz), although there was no evidence of a silent band that could facilitate sex recognition. Sex differences in call frequency also occurred within juveniles (*t*_16_ = 2.33, *p* < 0.05; female juvenile: 111.78 ± 1.84 kHz; male juvenile: 109.93 ± 1.54 kHz). No interaction was detected between population and sex, suggesting that the calls of both sexes responded in the same manner across localities.

A GLMM, in which population identity was modelled as a random factor, was fitted with all morphological and environmental variables. Of these, only relative humidity had a significant effect on call frequency (*t*_518_ = 3.846, *p* < 0.001). In addition, sex remained significant in the full model (*t*_518_ = 12.698, *p* < 0.001). We found no effect of elevation; however, *R. monoceros* is typically restricted to low elevations areas, and, although we sampled from a wide range of available elevations (20–460 m; mean, 230 m; s.d., 160.51 m), this might have limited the power of detecting an effect. A plot of humidity versus call frequency (figure 2) revealed that though variation in both variables co-varied, the relationship was not straightforward, with high call frequencies associated with low humidity in the south but with high humidity in the north. Indeed, separate correlations undertaken for call frequency against all three environmental variables were non-significant (data not shown).

### (b) Genetic structure, geographic distance and echolocation call frequency variation

**Figure 3.**
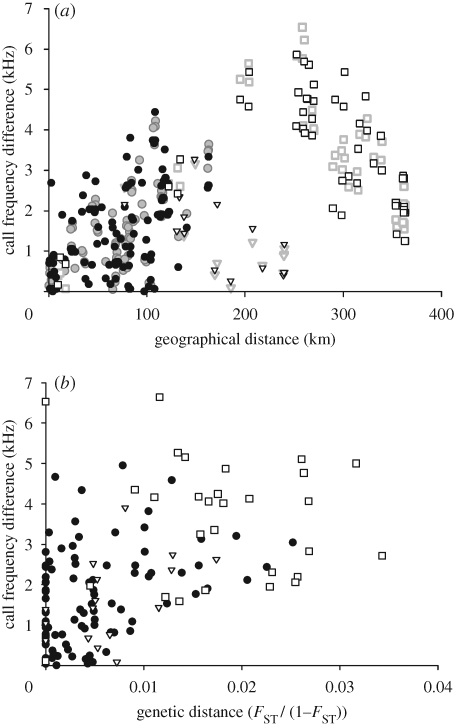
Pairwise call frequency difference versus (*a*) geographical divergence and (*b*) genetic distance in *Rhinolophus monoceros*. In both plots, solid circles denote comparisons among northerly populations (1–16), open triangles are comparisons between population 17 and other populations, and open squares are those between populations 18–20 and other populations. In (*a*) symbols are coloured grey for females and black for males.

For analyses of genetic structure, genotype data of females and males were pooled for each population, and only populations with sample sizes of over five were included. A plot of call frequency difference against geographical distance among pairs of populations approximated to a negative binomial distribution (figure 3*a*), although this appeared to be due to the combined effects of three sets of points, reflecting pairwise distances that included bats from (1) populations 1–16, (2) population 17, and (3) populations 18–20. These three groups broadly correspond to different clades in a mtDNA haplotype network with contrasting phylogeographic histories (Chen *et al.* 2006) and are also geographically separate (see figure 1). The overall pattern thus reflects the higher frequencies in the northern and southern populations and lower frequencies in the central areas (figure 2). Populations 1–16 (from the northern half and centre of Taiwan) exhibited a highly positive correlation between acoustic difference and geographical distance (females: *r*^2^ = 0.429, d.f. = 77, *p* < 0.01; males: *r*^2^ = 0.212, d.f. = 104, *p* < 0.01). In contrast, comparisons between populations 17–20 (from the south and southwest) and the more northerly populations showed a negative trend. The fact that the two main groups of pairwise differences (circles and squares) show positive and negative trends against geographical distance, respectively, reveals drift-like directionality in the change in call frequency over geographical distance within each region.

A plot of call frequency difference against pairwise genetic distance also showed a positive trend (*r*^2^ = 0.133, d.f. = 90, *p* < 0.01) (figure 3*b*); however, this was not significant after correcting for the effect of geographical distance (partial Mantel test, *r*^2^ = 0.057, *P* = 0.094). On the other hand, the correlation between geographical distance and call frequency difference remained significant after controlling for genetic distance (partial Mantel test, *r*^2^ = 0.301, *p* < 0.001).

The results of our cluster analyses revealed that the most likely number of multiple clusters, based on the outcomes of replicate runs, was *K* = 2. A monotonic decrease in likelihood values from *K* = 2 to *K* = 8 precluded the application of the Δ*K* method for further resolution of *K*. Of 20 independent runs for *K* = 2, ten were characterized by high pairwise similarity scores (>0.95), and were subsequently used to estimate average individual cluster membership for each population. This revealed that the two clusters of sampled populations of *R. monoceros* in Taiwan are broadly separated along a latitudinal gradient (see figure 1). Populations comprising individuals assigned mostly to cluster 2 were from the southwest (17), the far south (18, 19 and 20), and, to a lesser extent, the east (14–15). The eastern populations are northeast of a population (17) assigned to cluster 1, and thus appear to be outliers. However, these populations have previously been reported to show evidence of mixed genealogical origin involving the southern populations (Chen *et al.* 2006). The remaining populations comprised bats that were assigned mostly to cluster 1. These two clusters correspond directly to the two main groups of points in figure 3*a*. A plot of average cluster membership versus average call frequency per population confirms discontinuities between the southern populations and those from elsewhere in terms of both call frequency and cluster membership (see figure 4 in the electronic supplementary material).

## 4. Discussion

We recorded substantial variation in echolocation call frequency in *R. monoceros* both within and especially among populations. On average, females produced higher frequency calls and were larger than males. This trend, also seen in the morphologically similar *R. hipposideros* (Jones *et al.* 1992), runs counter to that across taxa, where body size correlates negatively with call frequency (Heller & von Helversen 1989; Francis & Habersetzer 1998). Although sex differences in call frequency have been reported in some other rhinolophids (Jones 1995; Russo *et al.* 2001), this is not universal (see Jones 1995). The marked overlap in frequency between males and females reported here suggests that call frequency is probably a poor cue for sex recognition (Jones 1995). We also found that call frequency was lower in juveniles than in adults. Jones & Ransome (1993) demonstrated that acquired vocal learning plays an important role in determining the final resting frequency in *R. ferrumequinum* offspring. Therefore, variation within populations may be attributable to postnatal learning, variation in proportions of sex and age classes, and by physiological differences among individuals.

Significant inter-population variation in call frequency associated with geographical distance shows remarkable similarities with the results of work on human language. Cavalli-Sforza & Wang (1986) drew parallels between linguistic change and gene replacement, and applied population genetics stepping stone models of isolation-by-distance to explore the relationship between geographical distance and lexical similarity. A resulting nonlinear positive relationship was attributed to variable rates of change across words. By comparison, the much simpler signal structure of rhinolophid CF echolocation calls correlates broadly linearly with geographical distance within regions. A similar trend between call frequency difference and genetic distance appeared to be an artifact resulting from the close association between genetic differentiation and geographical distance (Chen *et al.* 2006). When the effect of geographical distance was removed, the relationship between call frequency and genetic distance became non-significant. Thus it appears that call frequency difference and genetic distance co-vary with geographical distance, and that drift has an impact on both measures.

The relationship between human language similarity and geographical distance may also be disrupted by physical barriers to diffusion, which are expected to lead to greater vocal divergence (Cavalli-Sforza 2000). Our results show clear evidence that vocal isolation-by-distance in *R. monoceros* breaks down due to a change in the direction of drift in call frequency in the southern populations, which also coincides with a discontinuity in allele frequencies between this and other regions. Similar discontinuities in allele frequency within broadly continuous horseshoe bat populations have previously been shown to reflect suture zones between different refugial populations (Rossiter *et al.* 2007; Flanders *et al.* 2009). An earlier phylogeographic study of *R. monoceros* indicated that although the Taiwan population is monophyletic, the south has experienced both different demographic and evolutionary histories compared with other populations (Chen *et al.* 2006). Therefore, we suggest that concordant sharp discontinuities in allele and call frequencies reflect secondary contact following a long period of historical isolation, possibly related to climate change associated with past glaciation, in line with a vicariance-based model of acoustic variation.

Cultural drift is traditionally considered to be directional, whereas genetic drift is random. In our study, however, call frequencies in the south appear to be drifting in the opposite direction to those of their nearest sampled populations, and it is unclear whether this variation, and particularly the higher frequencies in both the north and south, are due to chance drift or are of adaptive significance. The frequency differences reported are unlikely to have consequences for diet or habitat use because they correspond to small differences in wavelength. Indeed, the recorded range of calls in *R. monoceros* corresponds to a range of wavelengths of 2.94–3.23 mm, assuming the speed of sound to be 345.67 m s^−1^ at a temperature of 22°C and a relative humidity of 80 per cent. It is therefore not meaningful to interpret the intra-specific variation in terms of partitioning diet by prey size (Russo *et al.* 2001). Although humidity explained some variance in call frequency, there was no clear correlation between these variables. Indeed, the unexpected association between high humidity and high call frequency in the northern populations suggests that this result was an artifact of the effect of population identity. These findings appear similar to those from the hipposiderid *Rhinonicteris aurantia*, where call frequency appears not to relate to humidity, but instead shows concordance with phylogenetic distinctiveness, and seems to be evolving in different directions among isolated populations (Armstrong & Coles 2007, and references therein).

Variation in call frequency among populations might also stem from social divergence. Work on birds and mammals supports the theory that cultural drift or social selection can restrain and maintain local vocal repertoires (Kingston *et al.* 2001; Wright & Wilkinson 2001). This hypothesis highlights the communication function of echolocation calls that have been demonstrated for recognition in mother–infant pairs (Matsumura 1981) and conspecifics (Leippert *et al.* 2000) and speculated for roost mates (Pearl & Fenton 1996) in various species of bat including *Rhinolophus*. A sex-recognition role for echolocation calls has also been posited, although this is based on stationary captive bats that might nevertheless be relevant to roosting wild bats (Kazial & Masters 2004). Given that the resting frequency of rhinolophoid calls is partly determined by vertical learning (Jones & Ransome 1993) and can be influenced by conspecifics (Hiryu *et al.* 2006), we suspect that social isolation is indeed important in hindering the homogenization of call frequencies among populations. The hypothesis that bat calls can shift in frequency to avoid overlap with co-distributed bat species, and so maintain a private bandwidth (Thabah *et al.* 2006; Russo *et al.* 2007), is also not relevant in Taiwan, where there are no other species calling at similar frequencies.

Drivers of geographical acoustic variation based on vicariant events, adaptation to environmental conditions and social selection are unlikely to be mutually exclusive but instead probably act in concert, with their relative importance varying across different spatial scales. For example, regional differences in call frequency in *R. monoceros* might have arisen by maternal transmission followed by cultural drift or selection during past periods of isolation, whereas smaller-scale population differences will be more dependent on the extent of local mixing as well as the nature of colonization. Yoshino *et al.* (2008) also recently proposed a maternal transmission with cultural drift hypothesis to explain geographical differences in the call frequency of *Rhinolophus cornutus* populations on Okinawa, Japan, in light of female-biased philopatry gene flow, greater nuclear gene flow and a small population size.

These emerging results highlight the need for further work on the relationship between bat echolocation calls and population history and, in particular, clarification of the correspondence between patterns of echolocation call design and genetic structure.
